# Optimum water supplement strategy to restore reed wetland in the Yellow River Delta

**DOI:** 10.1371/journal.pone.0177692

**Published:** 2017-05-23

**Authors:** Xuehong Wang, Dongjie Zhang, Bo Guan, Qing Qi, Shouzheng Tong

**Affiliations:** 1 Key Laboratory of Wetland Ecology and Environment, Northeast Institute of Geography and Agroecology, Chinese Academy of Sciences, Changchun, Jilin, P. R. China; 2 University of Chinese Academy of Sciences, Beijing, P. R. China; 3 Key Laboratory of Coastal Environmental Processes and Ecological Remediation, Yantai Institute of Coastal Zone Research (YIC), Chinese Academy of Sciences (CAS), Yantai, Shandong, P. R. China; The Education University of Hong Kong, HONG KONG

## Abstract

In order to supply optimum water to restore reed wetlands used for bird habitats, a field investigation and greenhouse experiment were conducted. Three water supplementation stages (early stage at 20 May, middle stage at 20 July and later stage at 20 September, respectively) and five depths (0, 10, 15, 20 and 35 cm over the surface, respectively) were established, with three replicates for each treatment combination. Reed growth characteristics (survival rate, height, density and biomass) and soil properties of field investigation and experiment were recorded to determine the impacts of water supplementation on reed wetland restoration. The field investigation showed that reeds in natural wetlands grow better than those in degraded wetlands and soil properties in degraded wetlands were significantly different from soils in natural wetlands. With freshwater supplementation, reed growth characteristics and soil properties greatly improved. As water depth increased, reed growth decreased gradually. Reeds grew best in shallow water depth (≦10cm) than in the greater flooding depths. Saturated soils with no standing water at the early stage of reed growth increased reed survival and water depth can be increased as the reeds grow. During the process of water supplementation, soil salinity was reduced significantly. Soil salinity was reduced dramatically at early and middle stages of reed growth, but it increased slightly at the later stage. Soil pH increased greatly during the experiment. Soil total nitrogen (TN) and total organic carbon (TOC) showed contrasting changes, with soil TN decreasing and TOC increasing. To best manage reed wetlands restoration, we suggest saturating wetland in the spring to stimulate reed germination, increasing surface water depth up to 15cm at the stage of reed rapid growth, and then reducing water depth during the later growth stage.

## Introduction

Coastal wetlands are well known not only for economic benefits but also for ecological functions that significantly benefit wildlife, especially birds. Many coastal wetlands serve as breeding grounds and nurseries for numerous species and stopovers on migratory routes for migratory birds [1]. However, due to both pressures of environmental and human activities, coastal wetlands have paid a high environmental price [2–3], with a decrease in wetland area and resources overexploitation. Coastal wetland restoration and habitats creation have become tools for many environmental agencies, parks, regions, states or NGOs, and there have been many developments in wetland restoration science and technology [4]. In general, successful habitats restoration depends on restoration of plant communities. This restoration can be challenging in tidal marshes because they are usually distributed along inundation (moisture) gradients and salinity in coastal areas.

Numerous documents have pointed out the interactions between plant growth, water table and soil salinity [5–7]. Water table depth and salinity gradients influenced plant growth and community distribution in delta wetlands [8–11]. They also affected plant stem length, number of branches, biomass or yield [12]. In addition, studies have shown that water table depth (groundwater and surface water) and seasonal flooding changes altered seeding survival rate, growth and biomass allocation, and may be a critical determinant of plant recruitment and occurrence in wetlands [13–14]. Effects of salinity include low plant diversity, low plant productivity and degraded habitats conditions [15–17]. Because fresh water inputs reduced soil salinity over time, therefore, flooding wetland with fresh water became an efficient method to restore coastal wetlands degraded by fresh water shortage and salinization.

The Yellow River Delta (YRD) is one of the most active deltas with significant land-ocean interactions in the world [18], and important habitats for migrating birds in the world due to its geographic position. Common reed in this delta has decreased by 17% from 1986 to 2001 due to lack of fresh water and increased salinization because of this decline, which threatened rare and endangered species in this region [19]. Restoration of common reed marsh has gained more attention recently in the YRD. Since 2002, fresh water depending on water and sediment regulation has been supplemented in the YRD once per year (mostly in June or July) to improve wetland functions and restore reed habitat for rare birds [10]. In this process, several methods have been set up to assess the annual environmental flow allocation [20]. The quality and distribution of water flows and their effects on common reed wetlands were evaluated by some ecologists and environmental scientists [5,19]. They not only pointed out the annual minimum environmental requirement water, but also proposed the minimum, moderate and optimum water requirement using a correlation analysis between the wetland biota and water regime [5]. However, all above researches were carried in healthy wetlands and does not describe water requirements for degraded and restored wetlands, and all study only focused on ground water and ignored requirement of habitats. During the process of Yellow River’s water and sediment regulation period, mass water was irrigated into the wetland at once, and this is harmful to many plants, even to common reed. While, details such as when and how deep to supplement fresh water were not examined in the process of wetland restoration. Therefore, a greenhouse-experiment and field investigation was carried out to flood common reed at different times of year and at varying depths to answer these questions. In addition, characteristics of reed growth and soil properties in natural and degraded reed wetlands were also investigated. The objectives of this study were to 1) evaluate the effects of flooding stage and surface water depth on reed growth and soil properties, and 2) propose an optimal water supplementation regime to restore reed wetlands in the YRD. Results of this study will guide common reed wetland restoration in the YRD and other areas which face similar environmental threats.

## Materials and methods

### Study site description

**Fig 1:**
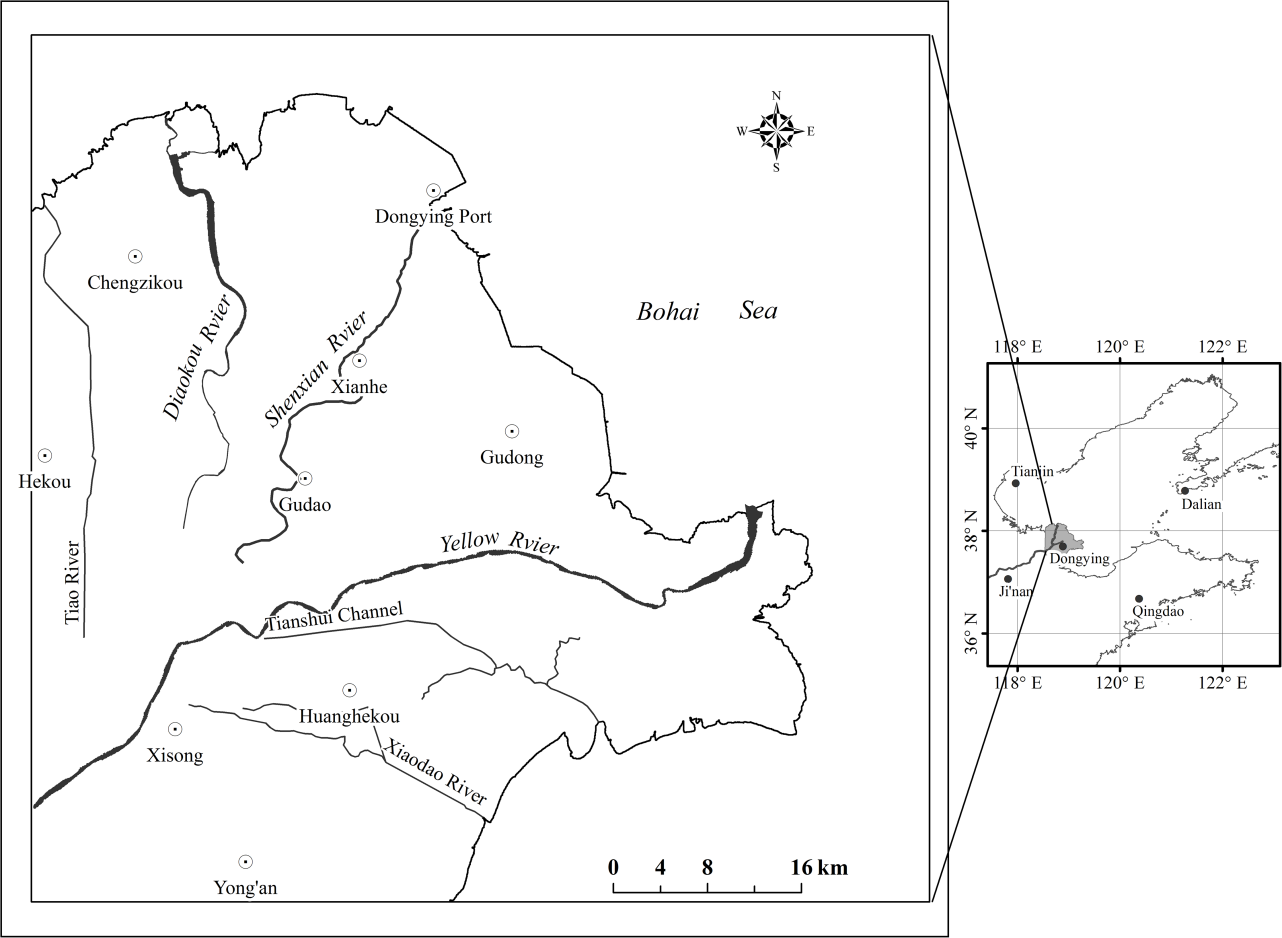
Study area of the Yellow River Delta.

The greenhouse experiment was conducted in the Shandong Key Laboratory for Eco-Environmental Science of Yellow River Delta (N37°22′52.34″, E117°58′45.65″) located in Shandong Province. Soil and reed rootstock were sampled from the tidal wetland of the Yellow River estuary (sample depth: 50cm, the soil electronic conductivity and pH is 3795.8±88.9 μs.cm^-1^ and 8.432±0.047, respectively, n = 15), which is located in the Nature Reserve of Yellow River Delta (YRDNR: E 118°33′~119°20′, N 37°35′~38°12′), Dongying City, Shandong Province, China (Fig 1). At the same time, a field investigation was also conducted at the YRDNR. The YRD is one of the most important habitats for migrating birds in the world and has great economic potential due to its geographic position. Because of regional development and global climate change, fresh water supplement decreased and substantial salinization aggravated in the region. The climate of YRDNR is warm temperate continental monsoon climate with distinctive seasons and rainy summer. The average temperatures in spring, summer, fall and winter are 10.7°C, 27.3°C, 13.1°C and-5.2°C, respectively.

### Ethics statement

This field investigation was carried out in the experimental zone of the YRDNR. The land of YRDNR is protected; however, researches activities conducted in the experimental zone did not need specific permission. In addition, field investigation in our study was conducted with the strict rules of the Yellow River Delta Nature Reserve Administration. During the process of field investigation, no protected species were sampled.

### Experimental methods

#### Greenhouse-experiment

Soil samples were poured into plastic pots (30 cm in diameter and 35cm in height). Reed roots were collected from multiple individuals in the study area at the beginning of experiment. All shoots were cleaned and cut to the same length (10cm). 12 shoots were planted in each pot and covered with 5 cm soil. Soils in all pots were kept saturated until they were moved to the pools to execute the experiment.

Based on field observations of habitat requirement, three flooding times and five flooding depths (FD) were chosen for this study. The flooding time included early stage (ES), middle stage (MS) and later stage (LS) of reed growth, which represents periods of germination/resume growth/seeding establishment, rapid growth and end of growth, respectively. Early stage started at 20 May, middle stage started at 20 July and later stage started at 20 September. Flooding depths were 0cm, 10cm, 15cm, 20cm, and 35cm. There were three replicates per treatment and 45 pots in total. Soils were kept saturated and all pots were kept their experiment status until the end of the experiment. The pots were watered regularly (3 days a time) with fresh water during the experiment. Reed growth characteristics were recorded at the end of experiment (20 October) and reeds were harvest and soils were sampled at the same time.

#### Field investigation

Based on pre-observations of vegetation, a fixed transect was set up in degraded and natural reed wetlands (total of 2 transects). 20 quads (1m×1m) were placed randomly along each transect. In October 2013, plant coverage, density and height in each quad were recorded and soil samples were collected.

### Data processing

Reed growth characteristics and soil properties in the greenhouse-experiment and field investigation were evaluated to describe effects of flooding stages and flooding depth on reed wetland restoration. Analysis was performed using SPSS. All data were examined (*p* = 0.05) using one-way analysis of variance (ANOVA) model for independent variables of flooding stage and flooding depth. Tukey’s HSD test was used to separate factors within these effects (*p* = 0.05).

## Results

### Reed growth characteristics and soil properties in natural and degraded wetland

Reed growth characteristics in natural and degraded wetland are shown in Table 1. Reeds in the natural wetland grew better than those in degraded wetland. ANOVA Results indicated there were significant differences between them. Soil bulk density and salinity increased, while content of TOC decreased. Soil bulk density, salinity and TOC showed obvious differences. Water content, TN and pH showed little changes without significant differences (Table 1) Correlation analysis indicated that biomass and height were significantly correlated with bulk density, water content, salinity and TOC (*p<0*.*05*), but not correlated with total nitrogen and pH.

**Table 1:** Reed characteristics and soil properties in natural and degraded reed wetlands.

		NW	DW
**Reed characteristics**	Height(cm)	122.69±1.61 a	70.6±3.35 b
	Density(stem/m^2^)	244±0.64 a	203.6±4.87 b
	Biomass(g/m^2^)	677.94±10.62 a	222.67±12.58 b
**Soil properties**	Bulk Density(g/cm^3^)	1.34±0.09 a	1.43±0.08 b
	Water Content (%)	29.31±4.27 a	28.83±4.81 a
	TOC(g/kg)	1.45±0.36 a	1.24±0.14 b
	TN(g/kg)	0.04±0.03 a	0.03±0.01 a
	EC(μC/cm)	1711.84±844.54 a	3536.83±893.28 b
	pH	8.81±0.07 a	8.83±0.23 a

NW: natural wetland; DW: degraded wetland. Different lower-case letters indicated differences in reed characteristics and soil properties in natural and degraded reed wetlands.

### Effects of water supplement on reed growth

**Fig 2:**
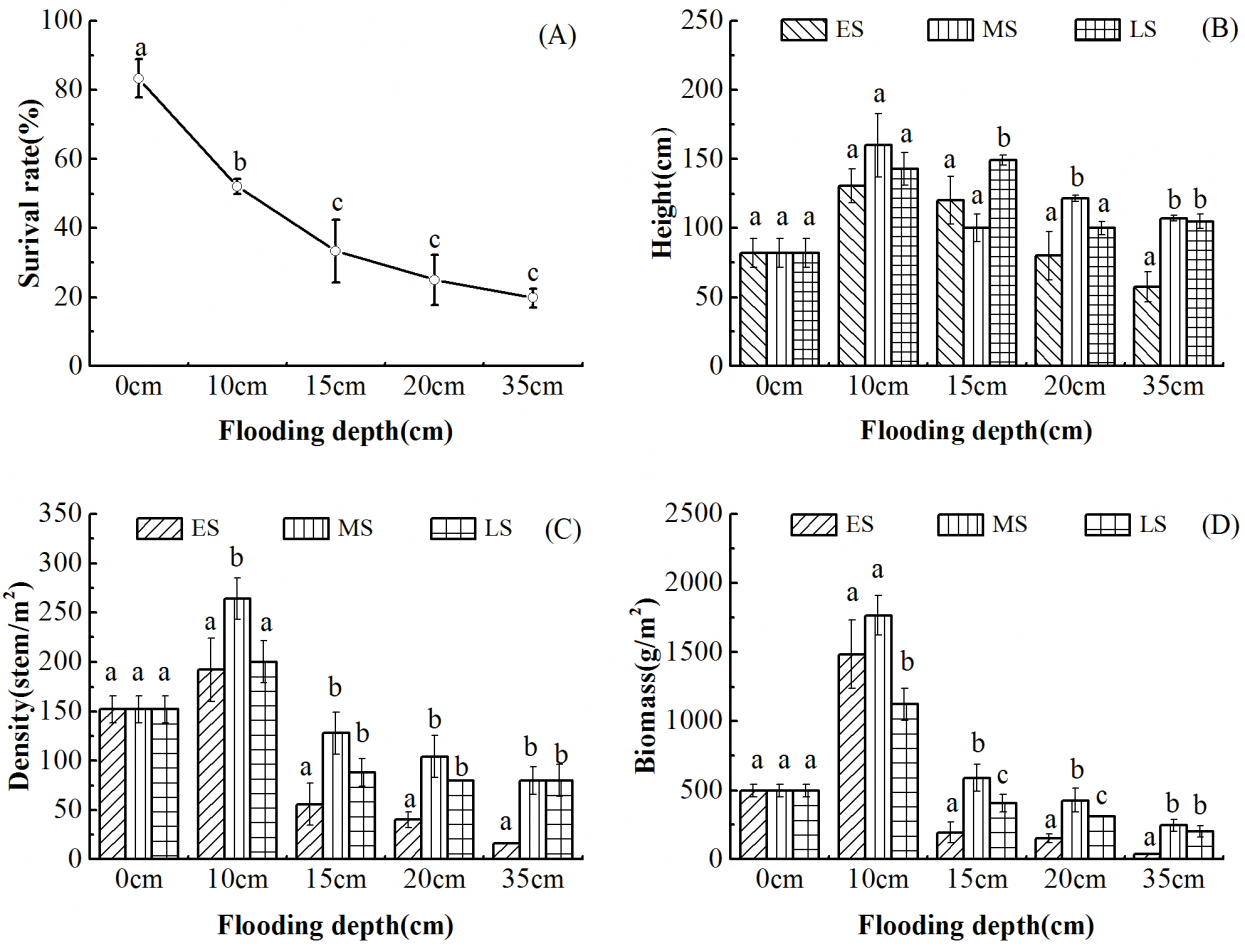
Effects of water supplement on reed survival (A), height (B), density (C) and biomass (D). Different lower-case letters indicated differences in reed survival (A), height (B), density (C) and biomass (D) subjected to different water depth.

Results showed that water supplement significantly affected reed growth (*p*<0.05). The first noticeable effect of an increase in surface water depth was a change in leaf color from light green to purple. In addition, more reeds decayed in deep-water. Survival rate of reed is best in saturated water (0 cm FD). Growth characteristics decreased with increasing flooding depths (Fig 2) and reeds subjected to 10 cm FD had the best growth characteristics. Flooding reed at different stage also indicated great differences. In the ES, shallow surface water depth, especially saturated (0 cm FD) condition contributed to reed seeding establishment and deep water restricted seedling survival. Reeds flooded in MS and LS were obvious taller than those flooded in ES. Density and biomass was the most in MS. The reason was that once reed seedlings were established, standing water, especially shallow surface water (about 10 cm FD) could promote reeds growth. In total, at each flooding stage, reed growth reduced once surface water is more or less than 10cm, and reed growth is best at every water depth at MS.

### Effects of water supplement on soil properties

**Fig 3:**
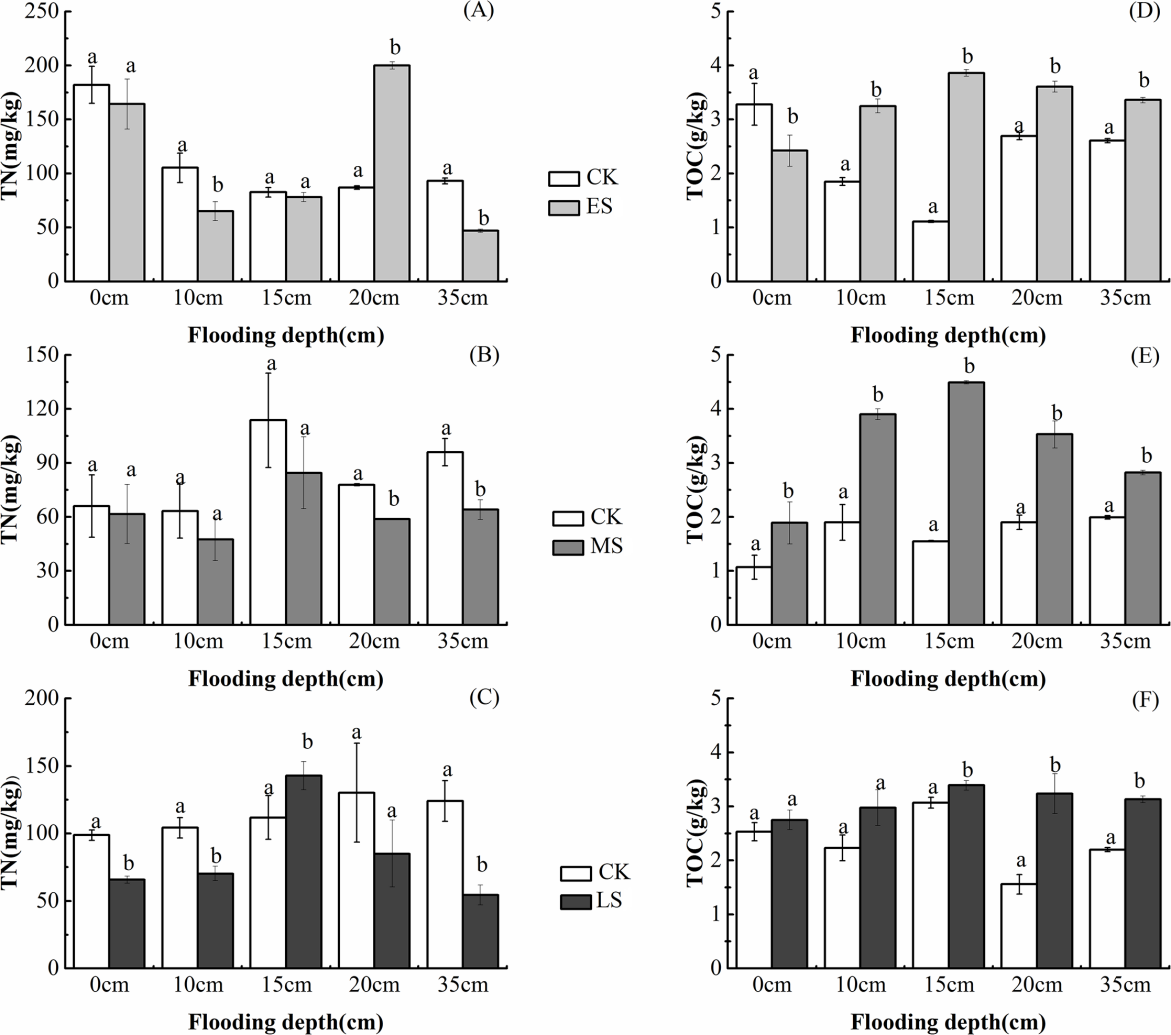
Total nitrogen (TN) and total organic carbon (TOC) subjected to different water supplement regimes: A-C is total nitrogen at early stage, middle stage and later stage; D-F is total organic carbon at early stage, middle stage and later stage. Different lower-case letters indicated differences in TN and TOC.

Water supplement also had great impacts on soil properties. Soil TN and TOC showed significant changes with a decrease in TN (Fig 3A–3C) and an increase in TOC (Fig 3D–3F) increased significantly. At each flooding stage and water depth, total organic carbon is higher than the control, and their differences are significantly (*p<0*.*05*). Meanwhile, compared with the control, total nitrogen decreased with surface water depth, except for at 20cm surface water depth at ES and 15cm surface water depth at LS. Differences analysis indicated that at ES, soil total nitrogen subjected to 10, 20 and 35cm surface water depth had a significant difference with the control. There were differences between TN subjected to 20 and 35 cm surface water depth and the control (*p<0*.*05*). While in LS, total nitrogen is different with the control, except for total nitrogen at 20 cm surface water depth.

**Fig 4:**
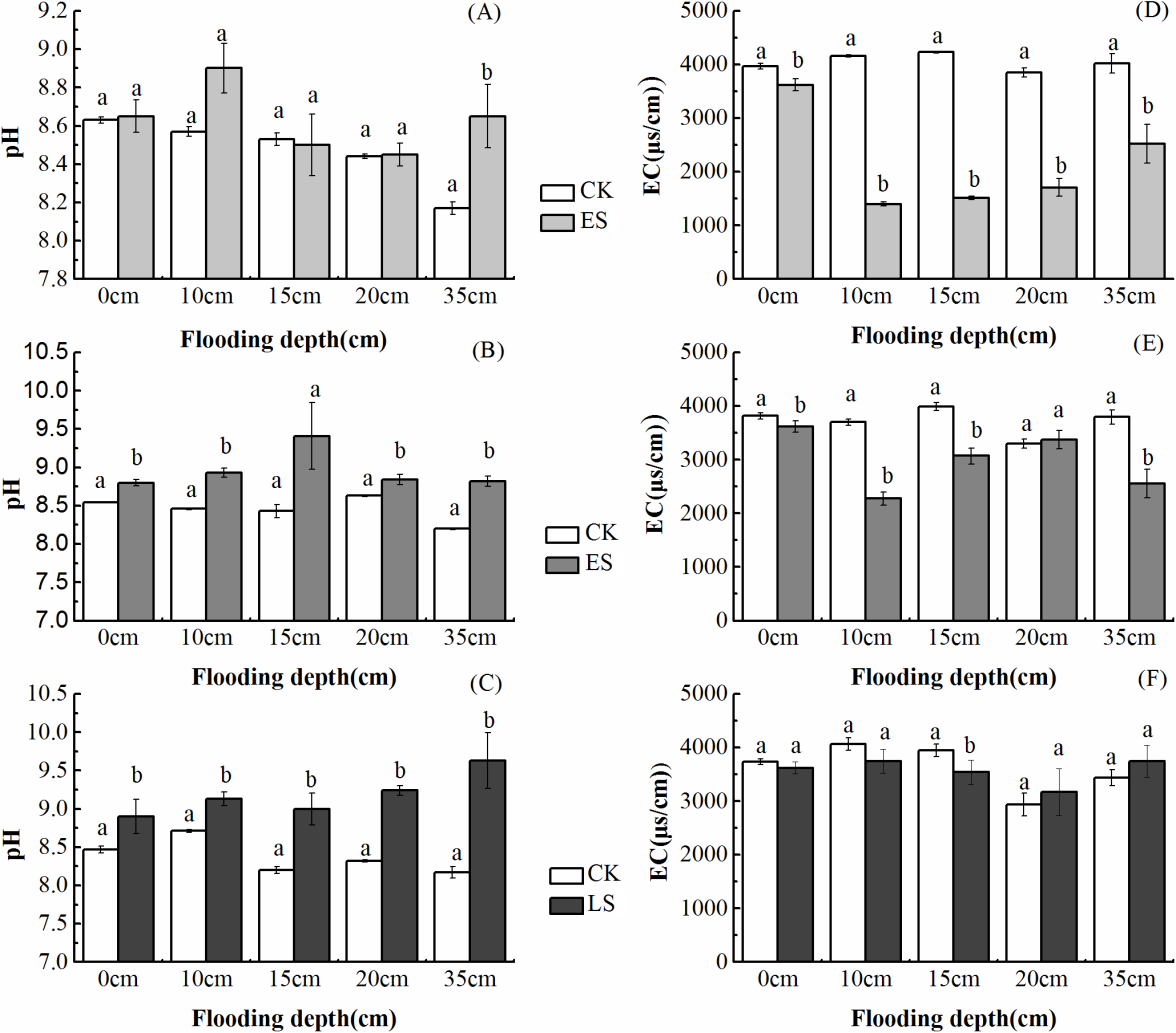
pH and electrical conductivity (EC) subjected to different water supplement regimes: A-C is pH at early stage, middle stage and later stage; D-F is electrical conductivity (EC) at early stage, middle stage and later stage. Different lower-case letters indicated differences in pH and EC.

Results of soil pH were interesting, without reduction, pH even increased greatly, but most pH is less than 9.0 (Fig 4A–4C). Soil salinity decreased during the process of water supplement (Fig 4D–4F). Salinity reduced dramatically in ES and MS, but increased lightly at LS. Compared with salinity in degraded wetlands, soil salinity has a notable reduction, but it still need time to be decreased to approach the salinity of natural reed wetland.

## Discussions

### Effects of water supplement on reed growth

Wetland vegetation could rapidly respond to changes in environmental conditions[4,21–22], such as increased flooding duration and water depth changes, and these effects were marked outcome through oxygen environment directly or indirectly. In deep water, *Myriophyllum spicatum* is taller with fewer and longer shoots [23]. Hellings and Gallagher (1992) demonstrated that shoot height and biomass decreased when water level increased [24]. Common reeds appeared to spread relatively fast in shallow water, but spread much slower when the water depth increased[25], while RGR and stem density was higher in shallow water than deep water[26], moderately fluctuating water levels could enhance reed growth[27]. Additionally, composition, distribution and growth characteristics of vegetation are mainly influenced by seasonal flooding and duration of inundation. Culm length of reed shows positively to water depth under static water but negatively under variable water [28], and seasonality flooding alter seeding survival, growth and biomass allocation, and this factor may be a critical determinant of plant recruitment and occurrence in wetlands [13].

In this study, reed grew better in natural wetland. Its biomass and height were affected significant by water content and salinity. With water content and surface water depth changes in the process of reed wetland degradation, oxygen condition changed correspondingly, and soil properties changed dramatically in soli salinity, bulk density and total organic carbon. All these resulted in degradation of reed growth. After water supplement, reed growth got improvement. Reed growth was the best under shallow surface water. Saturated water flooded in early stage is the best to promote reed seedling and establishment. Shallow surface water depth is the most suitable water depth for common reed in the middle stage of reed growth, and reed subjected to this condition indicated quick propagation. Water depth could be kept at a shallow surface depth from middle stage until the next spring, because reed grew enough strong and high to suffer a higher water flooded depth due to physiological features. This phenomenon will be studied systematically in the future.

### Effects of water supplement on soil properties

Water regime also influenced soil by controlling the extent and duration of saturation and it has a profound impact on salinity and other properties [29–30]. Soil salinity showed strong correlation with water regimes. With surface water and soil water changed, soil salinity varied in correspondingly. Salinity is well known to affect almost all aspects of plant and microorganism [31–32]. It suppresses seed germination, vegetation growth, plant biomass or yield and so on [33,34,35]. Low and moderate salinity would stimulate reed germination [36], while high salinity would constrict reed germination [37]. Vegetation patterns in salt marshes are also confined by their ecological thresholds for salinity [38]. Excessive amounts of salts cause adverse effects on soil physical and chemical properties [39–40], because salinity inhibits the degradation of organic matter, slow transformation of organic substrates and causes the accumulation of organic matter in the saline soil [41–43], and soil TC decreased along increasing salinity gradient. Water regime can also impact soil nutrient transfer. TN and TOC were significantly affected by water regime. The continuous water duration showed a higher total organic carbon and total nitrogen, as compared with the alternative wetting and drying. This phenomenon may be caused by Changes of oxidation-reduction potential which is influenced mainly by water regime. In the YRD, a study by Cui et al. (2009) who monitored soil salinity and soil organic matter in the YRD between 2001 and 2007, demonstrating reduced salinity and increased organic matter accumulation in soils with water supplementation [5], and this result is similar with our study. In our study, soil water content, TOC and TN decreased while bulk density, salinity and pH increased in the degraded wetlands. Particularly, soil salinity increased dramatically. After supplying freshwater to degraded reed wetlands, soil salinity and TN decreased while TOC increased significantly.

Up to now, a variety of research has been conducted on wetland restoration techniques and methods. Notwithstanding, there are still issues that need to be resolved, and coastal wetland restoration has a long way to go.

## Conclusions

In our study, common reed grew better in the natural wetland than that in the degraded wetland, and reed growth showed strong correlation with bulk density, water content, soil salinity and TOC. In the greenhouse-experiment, water supplementation significantly impacted reed growth and soil salinity. Most of all, there were water conservancy facilities to flood degraded reed wetland in the YRD, so it is possible to regulate flooding stage and surface water depth successfully. Therefore, we suggest that reed wetlands be flooded in the spring at the stage of reed germination or resume growth (at the end of April and begin of May), flood reed wetland at a lower mean flooding depth (≦10 cm FD). This flooding depth can stimulate germination or resuming of reed and increase its survival. At the stage of reed rapid growth (at the end of June and begin of July to the end of August), reeds require more water to support growth, so surface water depth could be increased up to 15cm.During this period, precipitation in this region is abundant, so water may be discharged to maintain reed growth. At the later stage of reed growth, reeds growing to mature have lower water requirement, so flooding depth may be decreased until the following spring. At each stage, excess water can be stored by existing water conservancy establishments to use for adjusting flooding water depths or water content as necessary.
